# The Community Structure of Picophytoplankton in Lake Fuxian, a Deep and Oligotrophic Mountain Lake

**DOI:** 10.3389/fmicb.2019.02016

**Published:** 2019-09-04

**Authors:** Xiaoli Shi, Shengnan Li, Huabing Li, Feizhou Chen, Qinglong Wu

**Affiliations:** ^1^State Key Laboratory of Lake Science and Environment, Nanjing Institute of Geography and Limnology, Chinese Academy of Sciences, Nanjing, China; ^2^Hunan Institute of Agro-Environment and Ecology, Hunan Academy of Agricultural Sciences, Changsha, China

**Keywords:** photosynthetic picoeukaryotes, picocyanobacteria, Lake Fuxian, community structure, seasonal succession

## Abstract

Spatial and seasonal dynamics of picophytoplankton were investigated by flow cytometry over a year in Lake Fuxian, a deep and oligotrophic mountain lake in southwest China. The contribution of picophytoplankton to the total Chl-*a* biomass and primary production were 50.1 and 66.1%, respectively. Picophytoplankton were mainly composed of phycoerythrin-rich picocyanobacteria (PE-cells) and photosynthetic picoeukaryotes (PPEs). PPEs were dominant in spring, reaching a maximum cell density of 3.0 × 10^4^ cell mL^–1^, while PE-cells were prevalent in other seasons. PE-cell abundance was relatively similar throughout the year, except for a decrease in summer during the stratification period, when nutrient concentration was low. High-throughput sequencing results from the sorted samples revealed that *Synechococcus* was the major PE-cell type, while Chrysophyceae, Dinophyceae, Chlorophyceae, Eustigmatophyceae, and Prymnesiophyceae were equally important PPEs. In spring, PPEs were mainly composed of Chlorophyceae and Trebouxiophyceae, while in summer, their dominance was replaced by that of Chrysophyceae and Prymnesiophyceae. Eustigmatophyceae and Chlorophyceae became the major PPEs in autumn, and Dinophyceae became the most abundant in winter. Single cells of *Microcystis* were usually detected in summer in the south, suggesting the deterioration of the water quality in Lake Fuxian.

## Introduction

Picophytoplankton (0.2–3 μm), comprising picocyanobacteria and photosynthetic picoeukaryotes (PPEs), are ubiquitous and important components of aquatic ecosystems. They contribute to 10–90% of the total plankton biomass and production in oceans and freshwaters (Stockner et al., 2000; Callieri, 2007). Picophytoplankton constitute an important energy resource in the aquatic microbial loop, and thus, are important in the biogeochemical processes (Cotner and Biddanda, 2002). Their contributions to the total biomass and production of plankton decline systematically with lakes of higher trophic status (Vörös et al., 1998; Bell and Kalff, 2001; Callieri et al., 2007). Environmental factors, such as water temperature, light limitation, nutrients and biotic factors, including grazing and viral-induced lysis, are all regarded as important factors controlling picophytoplankton abundances (Stockner and Shortreed, 1989; Lefranc et al., 2005; Lepère et al., 2006; Mózes et al., 2006; Callieri et al., 2012; Li et al., 2016).

The understanding of the taxonomy and dynamics of picophytoplankton is largely enriched due to the developments in molecular biology (Callieri, 2007; Lepère et al., 2010; Li et al., 2017; Zhang et al., 2019). *Synechococcus* is the most abundant photosynthetic prokaryotes living in oceans and lakes, and their relative abundance among the total autotrophic biomass increases with decreasing trophic state in aquatic systems (Bell and Kalff, 2001; Callieri, 2007). In fact, freshwater *Synechococcus* strains were polyphyletic and cannot be considered a natural taxon (Callieri, 2017). In contrast, studies regarding PPEs were mainly focused on marine ecosystems during recent decades and to a lesser extent in lacustrine environments (Li et al., 2017). In recent years, the combination of flow cytometric sorting and high-throughput sequencing has allowed progress in understanding the diversity and composition of PPEs in eutrophic shallow lakes (Li et al., 2017; Shi et al., 2018). However, the PPEs community composition in oligotrophic and deep lakes has rarely been investigated, especially in China.

In addition, previous studies related to picophytoplankton in freshwater lakes have mostly focused on temperate lakes. The seasonal cycle of picocyanobacteria populations has been studied in temperate lakes of all trophic types, and a diverse successional patterns have been noted (Weisse, 1988; Weisse and Kenter, 1991; Callieri and Stockner, 2002; Winder, 2009). There is relatively scarce information on the abundance and population dynamics of picophytoplankton in tropical freshwater systems. In fact, subtropical water ecosystems are different from temperate ecosystems in many aspects, e.g., temperature, light and the food web (Bonilla et al., 2016; Iglesias et al., 2017). The aim of the present study is to expand our knowledge of the abundance and composition of the picophytoplankton community in warm subtropical and deep oligotrophic lakes.

## Materials and Methods

### Study Site Description

Lake Fuxian is a subtropical and oligotrophic freshwater lake located in central Yunnan Province (24°17′-37′N, 102° 49′-57′E, altitude 1721 m, surface area 212 km^2^, volume 189 × 10^8^ m^3^). It is the second deepest lake in China, with maximum and average depths of 155 and 89.7 m, respectively. The water retention time of Lake Fuxian is ∼167 years. The annual average rainfall is 951.4 cm. Lake Fuxian is considered warm-monomictic, with mixing during the cool dry season and thermal stratification from May to September.

### Environmental Variables

**FIGURE 1:**
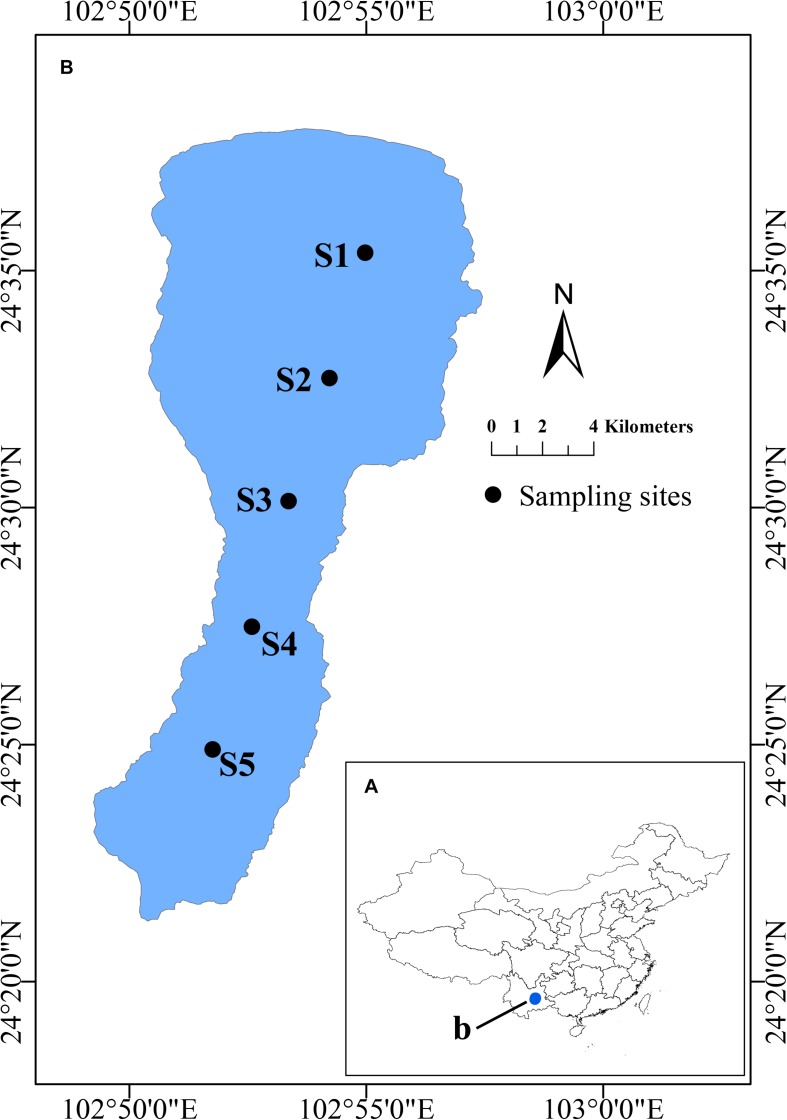
Location of Lake Fuxian in China and the sampling sites. **(A)** Location of Lake Fuxian in China. **(B)** Distribution of the sampling sites in Lake Fuxian.

Water samples from 50 cm depth were collected in March, July, October and December of 2015 at 5 sites along a N-S axis of the lake (Figure 1). Water temperature (T), pH, dissolved oxygen (DO), total dissolved solids (TDS), nephelometric turbidity units (NTU),conductivity (COND) and oxidation-reduction potential (ORP) were determined *in situ* using a multiparameter water quality probe (YSI 6600, Yellow Springs, OH, United States). A Secchi disk (SD) was used to measure the water transparency *in situ*. Water samples were collected in sterile bottles and transported immediately to the laboratory near shore on ice for further analysis. Total nitrogen (TN) and total phosphorous (TP) nitrate-nitrogen (NO_3_^–^-N), ammonium-nitrogen (NH_4_^+^-N), and orthophosphate (PO_4_^3–^-P), and dissolved organic carbon (DOC) were determined as described before (Li et al., 2016). Subsamples used for flow cytometric analysis were fixed with paraformaldehyde (1% final concentration, 10% PBS, pH 7.5), quick-frozen with liquid nitrogen, and then kept at −80°C until analysis.

### Pigment Analysis

Chlorophyll *a* (Chl-*a*) was estimated as a proxy of size-fractionated phytoplankton biomass. The large planktonic size fraction was firstly collected under a gentle vacuum on 3 μm Millipore Isopore^TM^ membrane filters (Merck Millipore Ltd., Tullagreen, Carrigtwohill Co., Cork, IRL). Subsamples of the filtrate representing the pico-planktonic size fraction were further filtered through 0.2 μm Millipore Isopore^TM^ membrane filters. The different size-fractionated biomass were summed to calculate the total phytoplankton biomass. The Chl-*a* of the membrane filters were extracted overnight in 90% acetone and determined spectrometrically as described in Yan et al. (2004). Water samples collected directly on GF/C glass fiber filters (1.2 μm pore size; Whatman, Maidstone, England, United Kingdom) were used for phycocyanin analysis, which were extracted in Tris buffer (0.05 M, pH 7.0) and measured spectrofluorometrically as described in Yan et al. (2004)

### Primary Production Measurement

Primary production was determined as described in Li et al. (2015). Briefly, the oxygen production based on light-dark bottle incubation was determined to represent the plankton production. The 22-mL Perkin-Elmer headspace vials (Jansson et al., 2012) were filled with either integrated water samples representing the whole phytoplankton or filtrates representing the pico-planktonic size fraction. The vials were then submerged in waters near shore and incubated for 4 h under light and dark conditions. A micro fiberoptic oxygen transmitter with an oxygen sensor (PreSens Micro TX3, Germany) was used to determine the oxygen concentrations at the start and end of the incubation. The gross community production was determined by calculating the difference between bottles, assuming respiration to be the same in the light and dark bottles. All analyses were performed in triplicates.

### Flow Cytometric Analysis

The frozen samples were thawed on ice and then filtered through a 48 μm pore-sized sieve as pretreatment to eliminate large particles (such as metazoan zooplankton and algal aggregates) and to avoid blocking the nozzle. A FACSJazz^SE^ flow cytometer (Becton Dickinson, United States) equipped with two lasers emitting at 488 nm and 640 nm, respectively, was used for the analysis. Full details of the discrimination and counting of different picophytoplankton groups can be found in Li et al. (2015). Two groups of picophytoplankton, photosynthetic picoeukaryotes (PPEs) and phycoerythrin-rich picocyanobacteria (PE-cells), were clearly distinguished by the flow cytometry. They were also detected frequently during the investigation (Supplementary Figure 1). PPEs were identified with higher forward scattering (FSC) signals and far-red autofluorescence from Chl-*a*, while PE-cells were with lower FSC and rich phycoerythrin (PE) fluorescence (Supplementary Figure 1). PPEs and PE-cells (100,000 to 150,000 cells) were sorted in enrichment mode directly into Eppendorf tubes containing 180 μL of lysis buffer (Tris-HCl, pH 8; EDTA-Na_2_ 2 mM; Triton X-100, 1.2%) (Marie et al., 2010; Shi et al., 2018). They were stored at −20°C until DNA extraction.

### DNA Extraction, PCR, and Pyrosequencing

DNA was extracted from the sorted samples using the DNeasy Blood and Tissue Extraction Kit (Qiagen, Germany) as modified by Marie et al. (2010). The V4 region of 16S rDNA was amplified using the cyanobacteria specific primers CYA 781R(a) 5′-GAC TAC TGG GGT ATC TAA TCC CAT T-3′ and CYA 781R(b) 5′-GAC TAC AGG GGT ATC TAA TCC CTT-3′. The V4 region of the 18S rDNA was amplified using the universal eukaryote primers Ek-NSF573 (5′-CGCGGTAATTCCAGCTCCA-3′) and Ek-NSR951 (5′-TTGGYRAATGCTTTCGC-3′) (Mangot et al., 2013). The amplicons were purified using the PCR purification kit (Agencourt AMPure XP, Beckman) following the manufacturer’s instructions. They were subjected to paired-end sequencing on an Illumina MiSeq platform. The subsequent sequence processing and taxonomic affiliation were described in Shi et al. (2019). Singletons were removed before further analysis. Sequences have been deposited at NCBI under BioProject number PRJNA534173.

### Data Analysis

All of the statistical analyses and visualizations were implemented in the R environment (version 3.2.1^1^). The relationships between the environmental variables and the abundances of the picophytoplankton groups were assessed by Spearman correlations. The sequence data were Hellinger-transformed before further multiple statistical analyses to decrease the effect of rare species (Legendre and Gallagher, 2001). The correlations between the environmental factors and PPE community composition were explored using a Redundancy analysis (RDA).

## Results

### Physical and Chemical Characteristics

The main physical and chemical parameters in Lake Fuxian were monitored in 4 months representing different seasons in 2015. The mean, minimum and maximum values recorded for each variable are listed in Table 1. As a typical subtropical lake, the water temperature of Lake Fuxian showed a small variation throughout the year, ranging between 15°C and 23°C. As Lake Fuxian is an oligotrophic lake, the nutrient concentrations are relatively low. The TP and TN concentrations were approximately 0.01 and 0.3 mg L^–1^, respectively.

**TABLE 1:** The key environmental factors among different seasons of Lake Fuxian in 2015.

	**Spring**	**Summer**	**Autumn**	**Winter**
T (°C)	15.1 ± 0.4	21.9 ± 2.8	20.7 ± 2.4	16.3 ± 0.0
pH	8.53 ± 0.02	8.33 ± 0.69	9.30 ± 0.38	8.98 ± 0.74
NTU	5.39 ± 3.21	30.71 ± 1.44	16.75 ± 2.34	23.53 ± 7.07
ORP (mV)	236.20 ± 70.08	50.30 ± 25.52	1.23 ± 11.75	−4.78 ± 16.27
Cond (mS cm^–1^)	0.27 ± 0.00	0.31 ± 0.01	0.30 ± 0.01	0.27 ± 0.00
DO (mg L^–1^)	9.77 ± 0.45	7.81 ± 1.22	6.80 ± 2.53	8.39 ± 0.06
DOC (PPM)	4.53 ± 1.75	15.86 ± 8.70	6.91 ± 1.69	6.96 ± 1.72
TN (mg L^–1^)	0.33 ± 0.11	0.22 ± 0.07	0.34 ± 0.07	0.39 ± 0.04
TP (mg L^–1^)	0.02 ± 0.00	0.01 ± 0.00	0.01 ± 0.00	0.02 ± 0.00
NO_3_-N (mg L^–1^)	0.06 ± 0.06	0.04 ± 0.04	0.10 ± 0.03	0.10 ± 0.06
PO_4_-P (μg L^–1^)	1.00 ± 0.58	3.22 ± 0.91	2.11 ± 0.65	3.83 ± 1.43
NH_4_-N (mg L^–1^)	0.04 ± 0.01	0.03 ± 0.01	0.06 ± 0.05	0.02 ± 0.01

*The values are listed as mean ± sd.*

### Picophytoplankton Biomass, Production and Abundances

**FIGURE 2:**
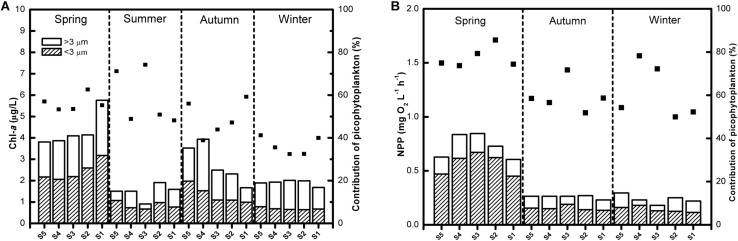
**(A)** The size fractioned Chl-*a* concentrations (the columns) and the contribution of picophytoplankton to total phytoplankton Chl-*a* (the points) in Lake Fuxian. **(B)** The size fractioned net primary production (NPP, the columns) and the contribution of picophytoplankton to total phytoplankton primary production (the points) in Lake Fuxian.

The total phytoplankton Chl-*a* concentration was quite low in Lake Fuxian, with the average level being 2.63 μg L^–1^. The Chl-*a* biomass of picophytoplankton was highest in spring, with an average of 2.47 μg L^–1^, whereas it maintained at a relatively low level in the other three seasons and reached its minimum in winter, with an average of 0.70 μg L^–1^ (Figure 2A). Likewise, the proportion of picophytoplankton to total phytoplankton Chl-*a* concentrations was lowest in winter, with an average of 36.3%, and highest in summer, with an average of 58.6% (Figure 1A). In addition, picophytoplankton also contributed greatly to the total phytoplankton primary production in Lake Fuxian (Figure 2B). Picophytoplankton production showed a similar dynamic with its Chl-*a* biomass and was highest in spring, with an average of 0.52 mg O_2_ L^–1^ h^–1^, followed by autumn and winter. Unfortunately, the primary production data in summer were missing due to a malfunction. The total phytoplankton primary production was clearly dominated by picophytoplankton, with its contributions being over 50% during most of the investigated year (Figure 2B).

**FIGURE 3:**
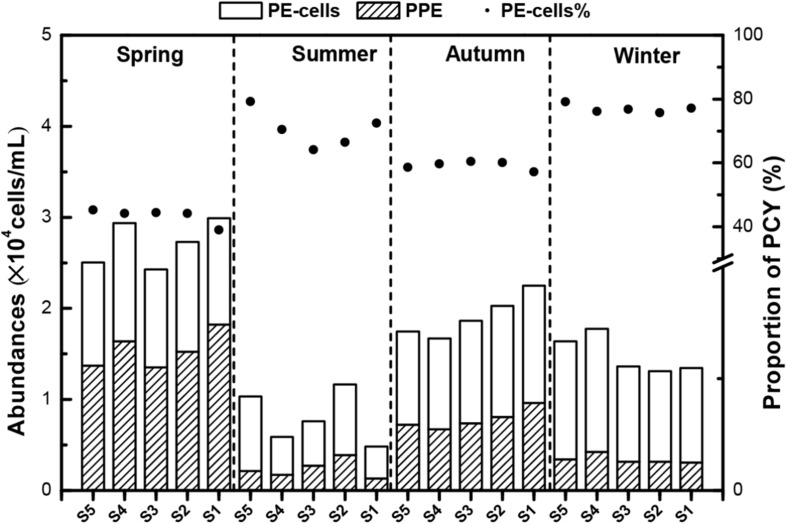
The abundances of photosynthetic picoeukaryotes (PPEs) and picocyanobacteria (PCY) in different seasons in Lake Fuxian. The points indicated the contributions of PCY to total picophytoplankton abundances.

Based on the flow cytometric scatter and fluorescence signals, two major picophytoplankton groups were identified in Lake Fuxian: PPEs and PE-cells (Supplementary Figure 1). These two groups were detected throughout the whole year at all sampling sites. The abundance of total picophytoplankton fluctuated throughout the year, and the highest levels were also achieved in spring, with approximately 3.0 × 10^4^ cells mL^–1^ (Figure 3). The dynamics of PE-cells and PPE abundances were similar to total abundances with concentrations ranging from 0.5 to 1.5 × 10^3^ cells mL^–1^ and 0.2 to 1.8 × 10^3^ cells mL^–1^, respectively. PE-cells dominated picophytoplankton in abundance in most of the seasons except in spring (Figure 3).

### Relationships Between Picophytoplankton Abundances and Environmental Factors

The Spearman correlation analyses showed that the abundances of PPEs and PE-cells showed significant relationships with many environmental variables (Table 2). Specifically, PPE abundance was significantly negatively correlated with PO_4_-P and positively correlated with Cond and DO concentrations, whereas PE-cell abundance showed little significance with those factors but was significantly positively correlated with TN and NO_3_-N concentrations. In addition, PPE and PE-cell abundances exhibited similar correlations with other environmental factors (Table 2).

**TABLE 2:** The correlations between environmental factors and the abundance of picophytoplankton.

	**PPEs**	**PE-cells**
T	−0.489,**	–0.539^∗∗^
pH	–0.172	0.135
ORP	–0.485^∗∗^	–0.607^∗∗^
Cond	0.768^∗∗^	0.262
DO	0.368^∗^	0.153
NTU	–0.865^∗∗^	–0.748^∗∗^
DOC	–0.453^∗∗^	–0.519^∗∗^
TN	0.201	0.572^∗∗^
TP	0.375^∗^	0.358^∗^
NO_3_-N	–0.047	0.370^∗^
PO_4_-P	–0.690^∗∗^	–0.293
NH_4_-N	0.284	0.28

*^∗^*P* value <0.01, ^∗∗^*P* value <0.05.*

### The Community Structure of Picocyanobacteria

**FIGURE 4:**
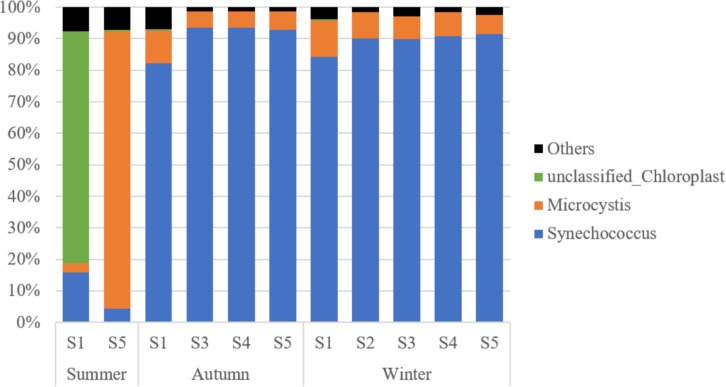
The taxonomic composition of PE-cells from the sorted samples in Lake Fuxian.

A total of 1,502,233 sequences were retrieved for the 16S rRNA from GenBank, over 99% of which were associated with cyanobacteria and grouped into 44 OTUs. *Synechococcus* turned out to be the most dominant cyanobacteria genus in Lake Fuxian, which accounted for 77.0% of the total cyanobacterial sequences. Unfortunately, the identification of cluster for the representative OTUs were not achieved, since the sequence of 400 bp is too short to build up a robust phylogenetic tree with high bootstrap values. In summer, at Station S1, 80.3% of the cyanobacteria sequences were related to unclassified Chloroplast, while only 18.1% of sequences belonged to *Synechococcus*. At Station S5, the majority of sequences were associated with *Microcystis*. In autumn and winter, more than 90% of cyanobacterial sequences were all linked to *Synechococcus* (Figure 4).

### The Community Structure of PPEs

**FIGURE 5:**
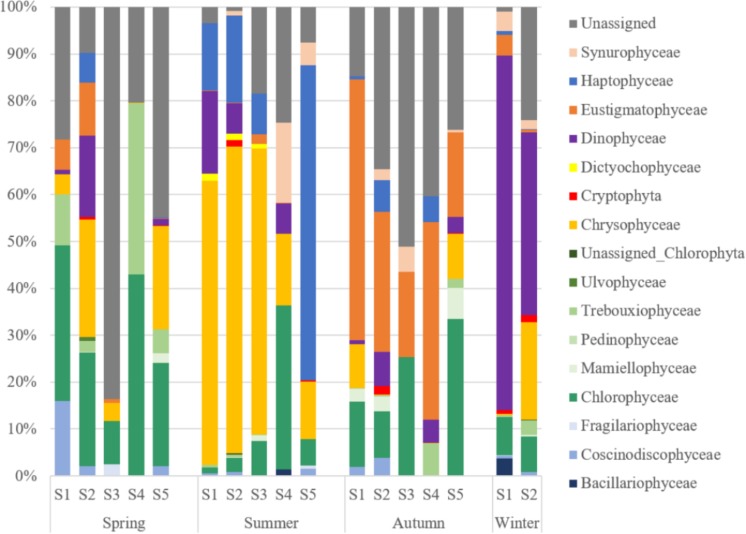
The taxonomic composition at class level of PPEs from the sorted samples in Lake Fuxian.

The diversity of PPEs was described based on a total of 730,293 quality-filtered reads grouped into 480 OTUs. Interestingly, the number of sequences affiliated with PPEs was quite low, and over 50% of the sequences were affiliated with non-pigmented picoeukaryotes. PPEs contributed to 37.87% of the total reads and represented only 165 OTUs. The taxonomic composition of the PPEs retrieved from Lake Fuxian is shown in Table 3. About 9.54% of the sequences were not assigned to any known PPE assemblages and were identified as unclassified PPEs in the present study. When compared at a high taxonomic level (i.e., class), Dinophyceae, Chrysophyceae, Chlorophyceae, Eustigmatophyceae, and Haptophyceae represented most of the PPE diversity in Lake Fuxian. In spring, PPEs were mainly composed of Chlorophyceae and Trebouxiophyceae, while in summer, their dominance was replaced by that of Chrysophyceae and Haptophyceae. Eustigmatophyceae and Chlorophyceae became the major PPEs in autumn, and Dinophyceae became the most abundant in winter (Figure 5).

**TABLE 3:** Taxonomic composition of putative photosynthetic picoeukaryotes retrieved in Lake Fuxian.

	**Numbers**		**Percentages (%)**	
	**OTUs**	**Reads**	**OTUs**	**Reads**
**Bacillariophyta**	**10**	**7377**	**2.08**	**1.01**
Bacillariophyceae	2	3279	0.42	0.45
Coscinodiscophyceae	7	2812	1.46	0.39
Fragilariophyceae	1	1286	0.21	0.18
**Chlorophyta**	**59**	**46555**	**12.29**	**6.37**
Chlorophyceae	38	37821	7.92	5.18
Mamiellophyceae	6	2371	1.25	0.32
Pedinophyceae	1	12	0.21	0.00
Trebouxiophyceae	8	6275	1.67	0.86
Ulvophyceae	3	19	0.63	0.00
Unassigned	3	57	0.63	0.01
**Chrysophyceae**	**43**	**75481**	**8.96**	**10.34**
**Cryptophyta**	**11**	**1303**	**2.29**	**0.18**
**Dictyochophyceae**	**4**	**1383**	**0.83**	**0.19**
**Dinophyceae**	**27**	**82869**	**5.63**	**11.35**
**Eustigmatophyceae**	**3**	**27259**	**0.63**	**3.73**
**Haptophyceae**	**2**	**27229**	**0.42**	**3.73**
**Synurophyceae**	**6**	**7128**	**1.25**	**0.98**
**Unassigned**	**22**	**69642**	**4.58**	**9.54**
**Sum**	**187**	**346226**	**38.96**	**47.41**

During the investigated year, the top 10 most abundant PPE OTUs detected in Lake Fuxian were mainly affiliated with Dinophyceae (OTU197, OTU194, OTU262, OTU267), Chlorophyceae (OTU203 and OTU23), Chrysophyceae (OTU80), Eustigmatophyceae (OTU14), Haptophyceae (OTU190) and Synurophyceae (OTU87). Specifically, the four dominant Dinophyceae OTUs were all affiliated with uncultured eukaryotes according to the BLAST results (Table 4). Except for Eustigmatophyceae OTU4, the other 5 dominant OTUs were all aligned to known species with high identity.

**TABLE 4:** Taxa of the 10 most abundant PPEs OTUs in Lake Fuxian, 2016.

**OTU ID**	**Reads (%)**	**Class**	**Blast closest relative (identity)**	**Accession number**
OTU80	8.38	Chrysophyceae	*Dinobryon sociale* (100%)	MK464020.1
OTU197	7.57	Dinophyceae	Uncultured eukaryote (99.71%)	JF317691.1
OTU190	3.73	Haptophyceae	*Chrysochromulina parva* (100%)	MH206612.1
OTU14	3.55	Eustigmatophyceae	*Eustigmatophyceae* sp. (87.29%)	KF757253.1
OTU203	1.64	Chlorophyceae	*Tetradesmus obliquus* (100%)	MK541731.1
OTU194	1.05	Dinophyceae	Uncultured eukaryote (100%)	JF317773.1
OTU262	0.80	Dinophyceae	Uncultured eukaryote (100%)	MG418714.1
OTU87	0.77	Synurophyceae	*Poterioochromonas malhamensis* (100%)	MH536661.1
OTU267	0.60	Dinophyceae	Uncultured eukaryote (99.71%)	JF317733.1
OTU233	0.51	Chlorophyceae	*Volvox aureus* (100%)	LC086362.1

### The Relationship of PPE Community Structure and Environmental Factors

**FIGURE 6:**
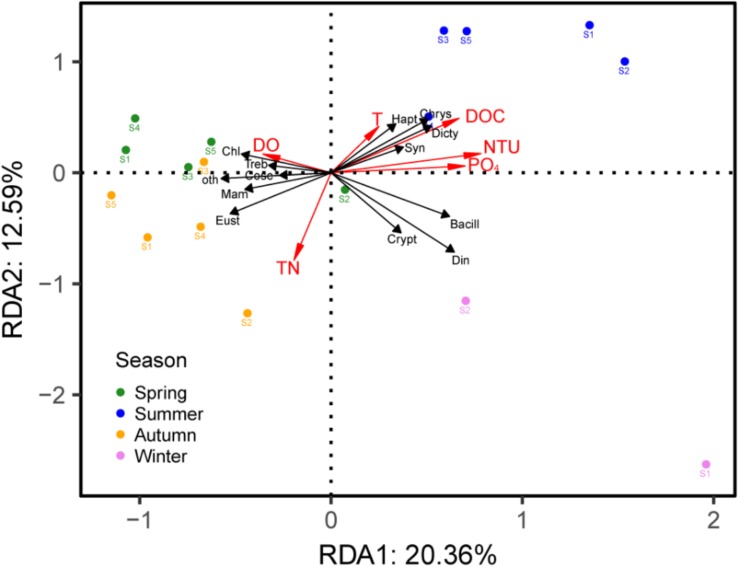
RDA triplots of samples, environmental parameters and the main taxa of PPEs in Lake Fuxian. Bacill, Bacillariophyceae; Cosc, Coscinodiscophyceae; Chl, Chlorophyceae; Mam, Mamiellophyceae; Treb, Trebouxiophyceae; Chrys, Chrysophyceae; Crypt, Cryptophyceae; Dicty, Dictyochophyceae; Din, Dinophyceae; Eust, Eustigmatophyceae; Hapt, Haptophyta; Syn, Synurophyceae; oth, other PPEs.

Based on the DCA analysis, the length of the first axis was 2.43; thus, we used the RDA analysis to investigate the correlation between the PPE communities and environmental factors, which showed a significant correlation (*p* = 0.008). After a forward selection based on the variance inflation factor (VIF) values, six factors, including DOC, TN, T, DO, NTU and PO_4_, were retained and entered into the model. The first two axes explained 20.36% and 12.59% of the PPE community variances, respectively, in Lake Fuxian (Figure 6). The results indicated that the samples from the same season tended to cluster together; specifically, spring and autumn samples were closer to each other and exhibited more similar PPE community compositions.

## Discussion

### The Contribution of Picophytoplankton in Lake Fuxian

Different populations of picophytoplankton in Lake Fuxian were identified based on their FSC (a proxy for cell size) signals and predominant pigments autofluorescence through flow cytometry. They were composed of phycoerythrin-rich prokaryotic and eukaryotic picophytoplankton. The relative proportion of picophytoplankton to total phytoplankton increases with decreasing trophic status of aquatic systems (Callieri, 2007; Ivanikova et al., 2007; Sterner, 2010; Shi et al., 2018). Unicellular picocyanobacteria and picoeukaryotes can outcompete the large phytoplankton in the ultraoligotrophic extreme of the trophic gradient (Suttle and Harrison, 1988; Suttle et al., 1988). One of the advantages of small cell size in low nutrient environments is that these organisms are less limited by molecular diffusion of nutrients because of the increase of the surface-to-volume ratio (Raven, 1998). Furthermore, better acclimation of picophytoplankton than that of the larger phytoplankton (>3 μm) to low-temperature and low-light winter environment was also confirmed by their higher maximum photosynthetic rate and light utilization parameter (Somogyi et al., 2016). Picophytoplankton concentrations in Lake Fuxian were in the 10^4^ cells mL^–1^ range during the investigated year, which were lower than that in the oligotrophic tropical lakes and eutrophic subtropical lakes, being in the 10^5^ cells mL^–1^ range (Sarmento et al., 2008; Li et al., 2016). The model of picophytoplankton contribution to total production in freshwater is largely based on results from a study of eight New Zealand lakes (Petersen, 1991). In a trophic gradient, expressed as increasing Chl-*a* concentration from 0.57–103 μg L^–1^, Petersen found an inverse relationship between picoplankton contribution to total carbon fixation and lake trophic state. In the present study in Lake Fuxian, which is an oligotrophic lake with low TN and TP concentrations, the mean contribution of picophytoplankton to total Chl-*a* biomass and primary production were 36.3% and 66.1%, respectively, which was significantly higher than that in the eutrophic lakes, Lake Taihu and Lake Chaohu (Li et al., 2016).

### Picocyanobacteria Dominate Picophytoplankton Abundances in Lake Fuxian

The present study showed that picocyanobacteria were prevalent in Lake Fuxian in most seasons. The prokaryotic structure of the picocyanobacteria cells provides them with the minimum costs for metabolism, and this factor has been considered as the main reason for their success in oligotrophic conditions (Weisse and Kenter, 1991). Besides, the ability to adapt to low-P conditions by accessing multiple forms of organic P also contributed to their success in oligotrophic conditions (Kutovaya et al., 2013; Callieri, 2017). In a 4-year study of picophytoplankton communities in Lake Maggiore, the abundance of picocyanobacteria gradually increased as the lake’s nutrient loads declined (Stockner, 1991). Lake typology and morphogenesis are also key factors influencing the picophytoplankton. Picocyanobacterial development was favored by the stability of the vertical structure of the lakes and by a high hydrological retention time. Large, deep lakes generally constitute preferred environments for the succession of picocyanobacteria (Stockner, 1991). In temperate lakes, the seasonal cycle of picocyanobacteria usually showed a bimodal pattern. They firstly peaked in spring or early summer, corresponding to the start of stratification, followed by a second peak during autumn (Stockner et al., 2000). In Lake Fuxian, picocyanobacteria showed equal abundances in winter as in spring and autumn because the temperature in winter is as high as 16°C in the subtropical region. The relatively low cell density in summer corresponded to the low nutrient concentration during the stratification period because our results showed that picocyanobacteria abundance was significantly positively correlated with TN and NO_3_-N concentrations.

The Chroococcales order has been considered as polyphyletic, with disperse clades among Cyanobacteria, based on phylogenetic analysis of the 16S rDNA sequences (Urbach et al., 1998). However, *Synechococcus* and *Cyanobium* are the two genera dominate the prokaryotic picophytoplankton in freshwater (Komárek, 1996). Our results indicated that *Synechococcus* was the major picocyanobacteria in Lake Fuxian in autumn and winter. Freshwater *Synechococcus* strains have developed the production of pigments to exploit various underwater light niches, thus being successful in different light fields along trophic gradients of lakes (Callieri, 2017). In summer, many sequences affiliated with *Microcystis*, which is a typical bloom species, were retrieved in Station S5. In fact, single *Microcystis* cells were also detected by flow cytometry in the late spring and autumn in the highly eutrophic lakes, Lake Chaohu and Lake Taihu (Li et al., 2016), and thus, the detection of *Microcystis* suggests a potential deterioration of water quality in Lake Fuxian. The water quality of Lake Fuxian is shown to be consistently good but is threatened by increasing pollution, as shown by the increasing trend of pollutants over the past 25 years (Chen et al., 2019). Since Lake Fuxian is designated as a drinking water conservation area, it is critical to take actions for pollution control.

### PPEs Community Structure in Lake Fuxian

Factors controlling PPE distribution differed markedly from those affecting picocyanobacteria both in space and time, largely because of their different nutritional and light requirements and potential growth rates (Weisse and Kenter, 1991). PPEs are often approximately one order of magnitude less abundant than picocyanobacteria, and in temperate regions, they tend to show a single population peak during spring isothermal mixing and early thermal stratification (Stockner, 1991; Fogg, 1995). Our results were consistent with this pattern, with PPEs in Lake Fuxian showing their peak abundances in spring.

To date, the community structure of PPEs in oligotrophic lakes has been little known. A previous study of Lake Pavin revealed that Chrysophyceae and Cryptophyta were major small pigmented eukaryotes (Lepère et al., 2006). However, classical methods using cloning and sequencing of the 18S rRNA genes in filtered water samples could cause an underestimation of PPEs because universal eukaryotic primers are heavily biased toward heterotrophs (Shi et al., 2009). Flow cytometric sorting has been proven to be a key advance in analyzing the PPEs community, and this approach produced a notable reduction in the contribution of heterotrophic groups within 18S rRNA gene clone libraries and allowed the recovery of several novel lineages (Shi et al., 2009).

Consistent with the presence of Chrysophyceae (Lepère et al., 2006), we also revealed the dominances of Dinophyceae, Eustigmatophyceae, Chlorophyceae and Haptophyceae in Lake Fuxian. In comparison, the PPE community structure was mainly composed of Chlorophyceae and Bacillariophyceae in eutrophic lakes (Li et al., 2017; Shi et al., 2018, 2019). Therefore, freshwater ecosystems with various trophic states contain different PPE community structures. Research from marine ecosystems also showed that PPE community structure can be quite different in regions with different trophic statuses. In oligotrophic water, Prasinophyceae (IX), clades of marine Chrysophyceae and Haptophyta dominated, whereas in the coastal region, groups with cultivated representatives such as *Mamiellales* prevailed (Shi et al., 2009). In addition, the community of PPEs in Lake Fuxian also exhibited a seasonal dynamic and were dominated by various taxa in different seasons. The RDA analysis indicated that the dominance of PPE taxa in different seasons was significantly correlated with environmental changes, which indicated that these PPE taxa can occupy different ecological niches.

The most abundant OTU in Lake Fuxian was affiliated with *Dinobryon sociale*. *Dinobryon* has been reported to be present in oligotrophic lakes and has high affinity for low ambient concentrations of inorganic phosphate and a capacity to absorb organically bound phosphate (Lehman, 1976; Dokulil and Skolaut, 1991). A bloom of *Dinobryon sociale* was recorded in Lake Balaton in 1993, caused by the release of resting cysts from sediment to the water by a coastal dredging operation (Reynolds et al., 1993). Four OTUs were associated with Dinophyceae, contributing approximately 10% of the total reads. Dinophyceae was widely present in the oligotrophic mountain lakes of the northern and southern slopes of the eastern Alps (Tolotti et al., 2003). Another dominant OTU belongs to *Chrysochromulina parva*. This is a common species in oligotrophic lakes that is mixotrophic and has potential toxicity (Parke et al., 1962; Hansen et al., 1994; Queimaliños, 2002). One potential advantage of mixotrophy is the acquisition of nitrogen and phosphorus from particulate food when concentrations of dissolved nutrients are low (Sanders et al., 2000). Consistent with our results, Eustigmatophyceae was reported to be a common member of the phytoplankton community in Lake Baikal and occurred throughout the year (Fietz et al., 2010). Chlorophyceae, including *Volvox aureus* and *Tetradesmus obliquus*, is widely distributed in the freshwater ecosystem, and the latter is considered a promising green microalgae for sustainable production of biofuels (Di et al., 2017).

In summary, picophytoplankton was a major contributor to phytoplankton biomass and primary production in Lake Fuxian, a deep and oligotrophic mountain lake in the southwest China. PPEs were dominant in spring, while phycoerythrin-rich *Synechococcus* was prevalent in other seasons. PPEs community composition exhibited a seasonal variation. In spring, PPEs were mainly composed of Chlorophyceae and Trebouxiophyceae, while in summer, their dominance was replaced by that of Chrysophyceae and Prymnesiophyceae. Eustigmatophyceae and Chlorophyceae became the major PPEs in autumn, and Dinophyceae became the most abundant in winter. Furthermore, single *Microcystis* cells were also detected in the lake in summer, suggesting the deterioration of the water quality in Lake Fuxian.

## Data Availability

The datasets generated for this study have been deposited atNCBI under BioProject number PRJNA534173.

## Author Contributions

XS conceived and designed the experiments. SL and HL performed the experiments. SL analyzed the data. FC and QW contributed reagents, materials, and analysis tools. XS and SL wrote the manuscript.

## Conflict of Interest Statement

The authors declare that the research was conducted in the absence of any commercial or financial relationships that could be construed as a potential conflict of interest.

## Footnotes

1 http://www.r-project.org

## References

[B1] Bell T. Kalff J. (2001). The contribution of picophytoplankton in marine and freshwater systems of different trophic status and depth. *Limnol. Oceanogr.* 46 1243–1248. 10.4319/lo.2001.46.5.1243

[B2] Bonilla S. Gonzalez-Piana M. Soares M. C. S. Huszar V. L. M. Becker V. Somma A. (2016). The success of the cyanobacterium *Cylindrospermopsis raciborskii* in freshwaters is enhanced by the combined effects of light intensity and temperature. *J. Limnol.* 75 606–617. 10.4081/jlimnol.2016.1479

[B3] Callieri C. (2007). Picophytoplankton in freshwater ecosystems: the importance of small-sized phototrophs. *Freshw. Rev.* 1 1–28. 10.1608/frj-1.1.1

[B4] Callieri C. (2017). Synechococcus plasticity under environmental changes. *FEMS Microbiol. Lett.* 364:fnx229 10.1093/femsle/fnx229 29092031

[B5] Callieri C. Cronberg G. Stockner J. G. (2012). “Freshwater picocyanobacteria: single cells, microcolonies and colonial forms,” in *Ecology of Cyanobacteria II*: *Their Diversity in Time and Space*, 2nd Edn, ed. Whitton B. A. (Berlin: Springer), 229–269. 10.1007/978-94-007-3855-3_8

[B6] Callieri C. Modenutti B. Queimalinos C. Bertoni R. Balseiro E. (2007). Production and biomass of picophytoplankton and larger autotrophs in Andean ultraoligotrophic lakes: differences in light harvesting efficiency in deep layers. *Aquat. Ecol.* 41 511–523. 10.1007/s10452-007-9125-z

[B7] Callieri C. Stockner J. G. (2002). Freshwater autotrophic picoplankton: a review. *J. Limnol.* 61 1–14. 15666689

[B8] Chen J. Lyu Y. Zhao Z. Liu H. Zhao H. Li Z. (2019). Using the multidimensional synthesis methods with non-parameter test, multiple time scales analysis to assess water quality trend and its characteristics over the past 25 years in the Fuxian Lake, China. *Sci. Total Environ.* 655 242–254. 10.1016/j.scitotenv.2018.11.144 30471592

[B9] Cotner J. B. Biddanda B. A. (2002). Small players, large role: microbial influence on biogeochemical processes in pelagic aquatic ecosystems. *Ecosystems* 5 105–121. 10.1007/s10021-001-0059-3

[B10] Di C. F. Pagnanelli F. Wijffels R. H. Van der Veen D. (2017). Quantification of Acutodesmus obliquus (Chlorophyceae) cell size and lipid content heterogeneity at single cell level. *J. Phycol.* 54 187–197. 10.1111/jpy.12610 29194643

[B11] Dokulil M. T. Skolaut C. (1991). Aspects of phytoplankton seasonal succession in Mondsee, Austria, with particular reference to the ecology of Dinobryon Ehrenb. *Internationale Vereinigung für Theoretische und Angewandte Limnologie: Verhandlungen* 24 968–973. 10.1080/03680770.1989.11898892

[B12] Fietz S. Bleiß W. Hepperle D. Koppitz H. Krienitz L. Nicklisch A. (2010). First record of *Nannochloropsis limnetica* (Eustigmatophyceae) in the autotrophic picoplankton from Lake Baikal. *J. Phycol.* 41 780–790. 10.1111/j.0022-3646.2005.04198.x

[B13] Fogg G. E. (1995). Some comments on picoplankton and its importance in the pelagic ecosystem. *Aquat. Microb. Ecol*. 9 33–39. 10.3354/ame009033

[B14] Hansen L. R. Kristiansen J. Rasmussen J. V. (1994). Potential toxicity of the freshwater *Chrysochromulina* species *C. parva* (Prymnesiophyceae). *Hydrobiologia* 287 157–159. 10.1007/bf00010731

[B15] Iglesias C. Meerhoff M. Johansson L. S. Gonzalez-Bergonzoni I. Mazzeo N. Pablo Pacheco J. (2017). Stable isotope analysis confirms substantial differences between subtropical and temperate shallow lake food webs. *Hydrobiologia* 784 111–123. 10.1007/s10750-016-2861-0

[B16] Ivanikova N. V. Popels L. C. McKay R. M. L. Bullerjahn G. S. (2007). Lake superior supports novel clusters of cyanobacterial picoplankton. *Appl. Environ. Microbiol.* 73 4055–4065. 10.1128/aem.00214-7 17468271 PMC1932735

[B17] Jansson M. Karlsson J. Jonsson A. (2012). Carbon dioxide supersaturation promotes primary production in lakes. *Ecol. Lett.* 15 527–532. 10.1111/j.1461-0248.2012.01762.x 22420750

[B18] Komárek J. (1996). Towards a combined approach for the taxonomy and species delimitation of picoplanktic cyanoprokaryotes. *Algol. Stud.* 83 377–401. 10.1127/algol_stud/83/1996/377

[B19] Kutovaya O. A. McKay R. M. L. Bullerjahn G. S. (2013). Detection and expression of genes for phosphorus metabolism in picocyanobacteria from the Laurentian Great Lakes. *J. Great Lakes Res.* 39 612–621. 10.1016/j.jglr.2013.09.009 19220397

[B20] Lefranc M. Thenot A. Lepere U. Debroas D. (2005). Genetic diversity of small eukaryotes in lakes differing by their trophic status. *Appl. Environ. Microbiol.* 71 5935–5942. 10.1128/aem.71.10.5935-5942.2005 16204507 PMC1266003

[B21] Lehman J. T. (1976). Ecological and nutritional studies on dinobryon ehrenb.: seasonal periodicity and the phosphate toxicity problem. *Limnol. Oceanogr.* 21 646–658. 10.4319/lo.1976.21.5.0646

[B22] Legendre P. Gallagher E. D. (2001). Ecologically meaningful transformations for ordination of species data. *Oecologia* 129 271–280. 10.1007/s004420100716 28547606

[B23] Lepère C. Boucher D. Jardillier L. Domaizon I. Debroas D. (2006). Succession and regulation factors of small eukaryote community. composition in a lacustrine ecosystem (Lake pavin). *Appl. Environ. Microbiol.* 72 2971–2981. 10.1128/aem.72.4.2971-2981.2006 16598004 PMC1449018

[B24] Lepère C. Masquelier S. Mangot J.-F. Debroas D. Domaizon I. (2010). Vertical structure of small eukaryotes in three lakes that differ by their trophic status: a quantitative approach. *ISME J.* 4 1509–1519. 10.1038/ismej.2010.83 20574460

[B25] Li S. Bronner G. Lepère C. Kong F. Shi X. L. (2017). Temporal and spatial variations in the composition of freshwater photosynthetic picoeukaryotes revealed by MiSeq sequencing from flow cytometry sorted samples. *Environ. Microbiol.* 19 2286–2300. 10.1111/1462-2920.13724 28276185

[B26] Li S. Shi X. Lepère C. Liu M. Wang X. Kong F. (2016). Unexpected predominance of photosynthetic picoeukaryotes in shallow eutrophic lakes. *J. Plankton Res.* 38 830–842. 10.1093/plankt/fbw042

[B27] Li S. Zhou J. Wei L. Kong F. Shi X. (2015). The effect of elevated CO2 on autotrophic picoplankton abundance and production in a eutrophic lake (Lake Taihu, China). *Mar. Freshw. Res.* 67 3179–3190.

[B28] Mangot J. F. Domaizon I. Taib N. Marouni N. Duffaud E. Bronner G. (2013). Short-term dynamics of diversity patterns: evidence of continual reassembly within lacustrine small eukaryotes. *Environ. Microbiol.* 15 1745–1758. 10.1111/1462-2920.12065 23297806

[B29] Marie D. Shi X. L. Rigaut-Jalabert F. Vaulot D. (2010). Use of flow cytometric sorting to better assess the diversity of small photosynthetic eukaryotes in the English channel. *FEMS Microbiol. Ecol.* 72 165–178. 10.1111/j.1574-6941.2010.00842.x 20236325

[B30] Mózes A. Présing M. Vörös L. (2006). Seasonal dynamics of picocyanobacteria and picoeukaryotes in a large shallow lake (Lake Balaton, Hungary). *Int. Rev. Hydrobiol.* 91 38–50. 10.1002/iroh.200510844

[B31] Parke M. Lund J. W. G. Manton I. (1962). Observations on the biology and fine structure of the type species of *Chrysochromulina* (*C. parva* Lackey) in the English Lake District. *Arch. Mikrobiol.* 42 333–352. 10.1007/bf00409070 14483908

[B32] Petersen R. (1991). Carbon-14 uptake by picoplankton and total phytoplankton in Eight New Zealand Lakes. *Int. Rev. Hydrobiol.* 76 631–641. 10.1002/iroh.19910760413

[B33] Queimaliños C. (2002). The role of phytoplanktonic size fractions in the microbial food webs in two north Patagonian lakes (Argentina). *Internationale Vereinigung für theoretische und angewandte Limnologie: Verhandlungen* 28 1236–1240. 10.1080/03680770.2001.11902651

[B34] Raven J. A. (1998). The twelfth tansley lecture. small is beautiful: the picophytoplankton. *Funct. Ecol.* 12 503–513. 10.1046/j.1365-2435.1998.00233.x

[B35] Reynolds C. S. Padisák J. Kóbor I. (1993). A localized bloom of *Dinobryon sociale* in Lake Balaton: some implications for the perception of patchiness and the maintenance of species richness. *Abstracta Bot.* 17 251–260.

[B36] Sanders R. W. Berninger U. G. Lim E. L. Kemp P. F. Caron D. A. (2000). Heterotrophic and mixotrophic nanoplankton predation on picoplankton in the Sargasso Sea and on Georges Bank. *Mar. Ecol. Prog.* 192 103–118. 10.3354/meps192103

[B37] Sarmento H. Unrein F. Isumbisho M. Stenuite S. Gasol J. M. Descy J. P. (2008). Abundance and distribution of picoplankton in tropical, oligotrophic Lake Kivu, eastern Africa. *Freshw. Biol.* 53 756–771. 10.1111/j.1365-2427.2007.01939.x

[B38] Shi X. Li S. Fan F. Zhang M. Yang Z. Yang Y. (2019). Mychonastes dominates the photosynthetic picoeukaryotes in Lake Poyang, a river-connected lake. *FEMS Microbiol. Ecol.* 95:fiy211. 10.1093/femsec/fiy211 30346526

[B39] Shi X. Li S. Liu C. Zhang M. Liu M. (2018). Community structure of photosynthetic picoeukaryotes differs in lakes with different trophic statuses along the middle-lower reaches of the Yangtze River. *FEMS Microbiol. Ecol.* 94:fiy011. 10.1093/femsec/fiy011 29360960

[B40] Shi X. L. Marie D. Jardillier L. Scanlan D. J. Vaulot D. (2009). Groups without cultured representatives dominate eukaryotic picophytoplankton in the oligotrophic South East Pacific Ocean. *PLoS One* 4:e7657. 10.1371/journal.pone.0007657 19893617 PMC2764088

[B41] Somogyi B. Felföldi T. Katalin V.-B. Boros E. Pálffy K. Vörös L. (2016). The role and composition of winter picoeukaryotic assemblages in shallow Central European great lakes. *J. Great Lakes Res.* 42 1420–1431. 10.1016/j.jglr.2016.10.003

[B42] Sterner R. W. (2010). In situ-measured primary production in Lake Superior. *J. Great Lakes Res.* 36 139–149. 10.1016/j.jglr.2009.12.007

[B43] Stockner J. Callieri C. Cronberg G. (2000). “Picoplankton and other non-bloom-forming cyanobacteria in lakes,” in *The Ecology of Cyanobacteria*, eds Whitton B. A. Potts M. (Dordrecht: Kluwer Academic Publishers), 195–231. 10.1007/0-306-46855-7_7

[B44] Stockner J. G. (1991). Autotrophic picoplankton in freshwater ecosystems: the view from the summit. *Internationale Revue der gesamten Hydrobiologie und Hydrographie* 76 483–492. 10.1002/iroh.19910760402

[B45] Stockner J. G. Shortreed K. S. (1989). Algal picoplankton production and contribution to food-webs in oligotrophic British Columbia lakes. *Hydrobiologia* 173 151–166. 10.1007/bf00015525

[B46] Suttle C. A. Harrison P. J. (1988). Ammonium and phosphate uptake kinetics of size-fractionated plankton from an oligotrophic freshwater lake. *J. Plankton Res.* 418 343–346.

[B47] Suttle C. A. Stockner J. G. Shortreed K. S. Harrison P. J. (1988). Time-courses of size-fractionated phosphate uptake: are larger cells better competitors for pulses of phosphate than smaller cells? *Oecologia* 74 571–576. 10.1007/BF00380055 28311764

[B48] Tolotti M. Thies H. Cantonati M. (2003). Flagellate algae (Chrysophyceae, Dinophyceae, Cryptophyceae) in 48 high mountain lakes of the Northern and Southern slope of the Eastern Alps: biodiversity, taxa distribution and their driving variables. *Hydrobiologia* 502 331–348. 10.1007/978-94-017-2666-5_27

[B49] Urbach E. Scanlan D. J. Distel D. L. Waterbury J. B. Chisholm S. W. (1998). Rapid diversification of marine picophytoplankton with dissimilar light-harvesting structures inferred from sequences of *Prochlorococcus* and *Synechococcus* (Cyanobacteria). *J. Mol. Evol.* 46 188–201. 10.1007/pl00006294 9452521

[B50] Vörös L. Callieri C. Balogh K. V. Bertoni R. (1998). Freshwater picocyanobacteria along a trophic gradient and light quality range. *Hydrobiologia* 370 117–125. 10.1007/978-94-017-2668-9_10

[B51] Weisse T. (1988). Dynamics of autotrophic picoplankton in lake constance. *J. Plankton Res.* 10 1179–1188. 10.1093/plankt/10.6.1179

[B52] Weisse T. Kenter U. (1991). Ecological characteristics of autotrophic picoplankton in a prealpine lake. *Int. Rev. Hydrobiol.* 76 493–504. 10.1002/iroh.19910760403

[B53] Winder M. (2009). Photosynthetic picoplankton dynamics in Lake Tahoe: temporal and spatial niche partitioning among prokaryotic and eukaryotic cells. *J. Plankton Res.* 31 1307–1320. 10.1093/plankt/fbp074

[B54] Yan R. Kong F. Han X. (2004). Analysis of the recruitment of the winter survival algae on the sediments of Lake Taihu by fluorometry. *J. Lake Sci.* 16 164–169. 10.18307/2004.0210

[B55] Zhang H. Feng J. Chen S. Zhao Z. Li B. Wang Y. (2019). Geographical patterns of nirS gene abundance and nirS-type denitrifying bacterial community associated with activated sludge from different wastewater treatment plants. *Microb. Ecol.* 77 304–316. 10.1007/s00248-018-1236-7 30046860

