# Supplementary material for: The Community Structure of Picophytoplankton in Lake Fuxian, a Deep and Oligotrophic Mountain Lake
Source: Front Microbiol. 2019 Sep 4;10:2016. doi: 10.3389/fmicb.2019.02016 (PMC6737998; doi:10.3389/fmicb.2019.02016)
Supplement: Supplementary file 1 [file Table_1.docx]

Supplementary Material

# Supplementary Figures


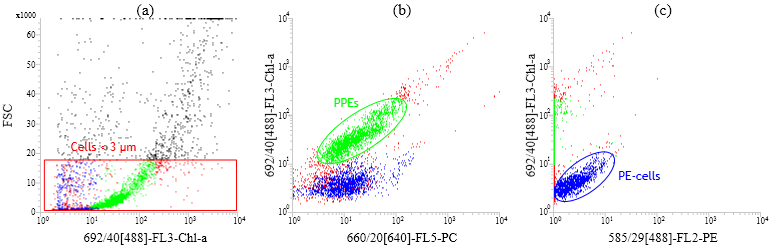


**Supplementary Figure 1.** The picophytoplankton groups identified by flow cytometry. (a) Cytogram of Chl-a fluorescence versus forward side scatter (FSC, a proxy for cell size). (b) Cytogram of PC fluorescence versus Chl-a fluorescence. (c) Cytogram of PE fluorescence versus Chl-a fluorescence.
